# Supplementary material for: Ni80Fe20 nanotubes with optimized spintronic functionalities prepared by atomic layer deposition
Source: Nanoscale. 2021 Jul 29;13(31):13451–62. doi: 10.1039/d1nr02291a (PMC8359140; doi:10.1039/d1nr02291a)
Supplement: NR-013-D1NR02291A-s001 [file NR-013-D1NR02291A-s001.pdf]

Electronic Supplementary Material (ESI) for Nanoscale.
This journal is © The Royal Society of Chemistry 2021

Supplementary information

$Ni_{80}Fe_{20}$ Nanotubes with Optimized Spintronic Functionalities Prepared by Atomic Layer Deposition

M. C. Giordano[1], S. R. Escobar Steinvall[2], S. Watanabe[1], A. Fontcuberta i Morral[2,3], and D. Grundler[1,4]

[1] Institute of Materials, Laboratory of Nanoscale Magnetic Materials and Magnonics, Ecole Polytechnique Federale de Lausanne, School of Engineering, 1015 Lausanne, Switzerland
[2] Institute of Materials, Laboratory of Semiconductor Materials, Ecole Polytechnique Federale de Lausanne, School of Engineering,1015 Lausanne, Switzerland
[3] Institute of Physics, School of Natural Sciences, Ecole Polytechnique Federale de Lausanne, 1015 Lausanne, Switzerland
[4] Institute of Microengineering, School of Engineering, Ecole Polytechnique Federale de Lausanne, 1015 Lausanne, Switzerland

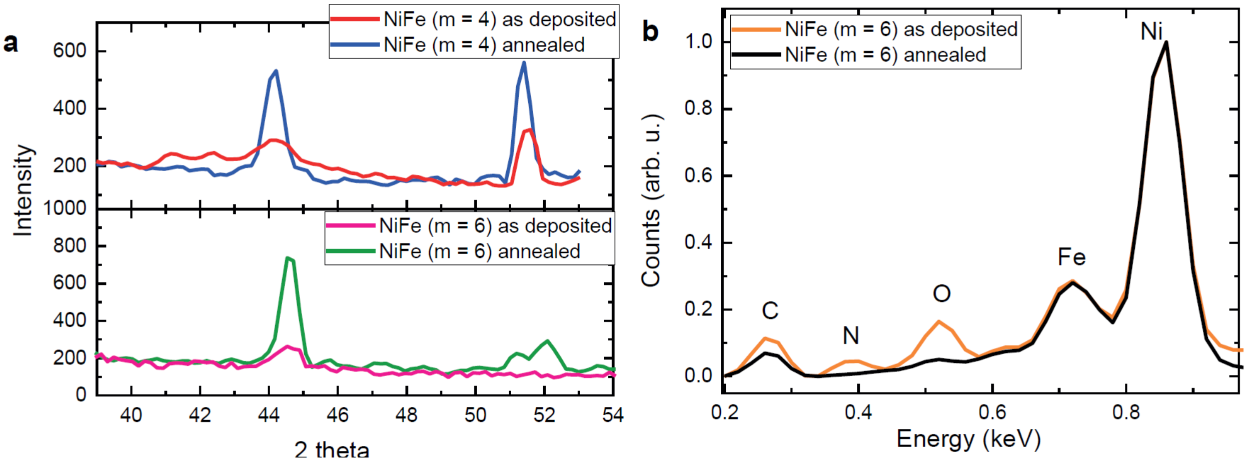

Figure S1. (a) X-Ray diffractograms of the samples prepared with *m* = 4 and *m* = 6 in their as deposited and annealed state. (b) Energy dispersive spectroscopy of the NiFe sample prepared with *m* = 6 as deposited and annealed in the energy range 0.2-1 keV. The annealing treatment was performed at 380 ˚C.

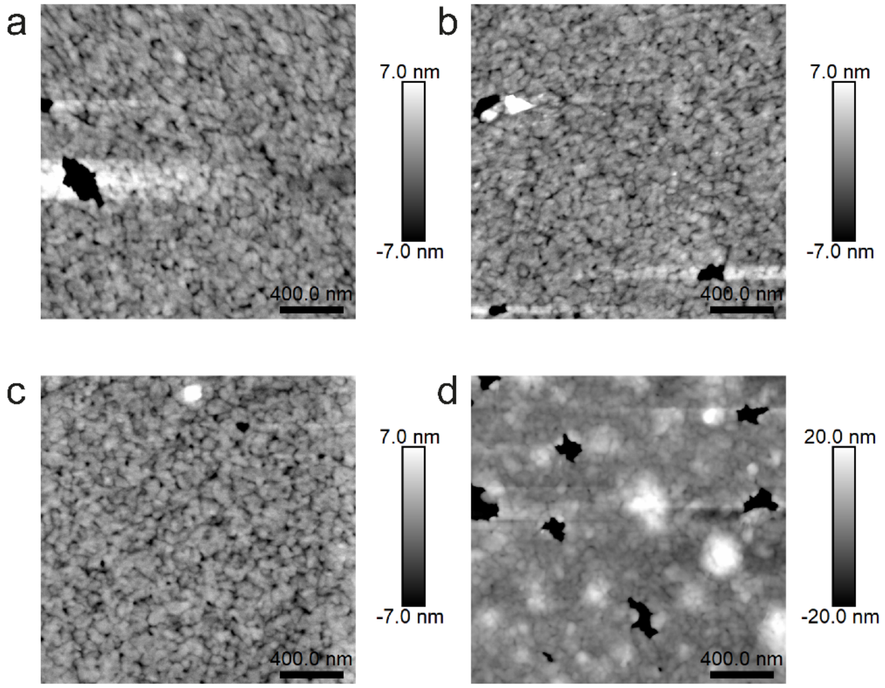

Figure S2. Atomic force microscopy performed on a 2 μm × 2 μm area of samples prepared with (a) m = 4, (b) m = 5, (c) m = 6, and (d) m = 7 on bare silicon substrates, after the annealing treatment at 380 ˚C.

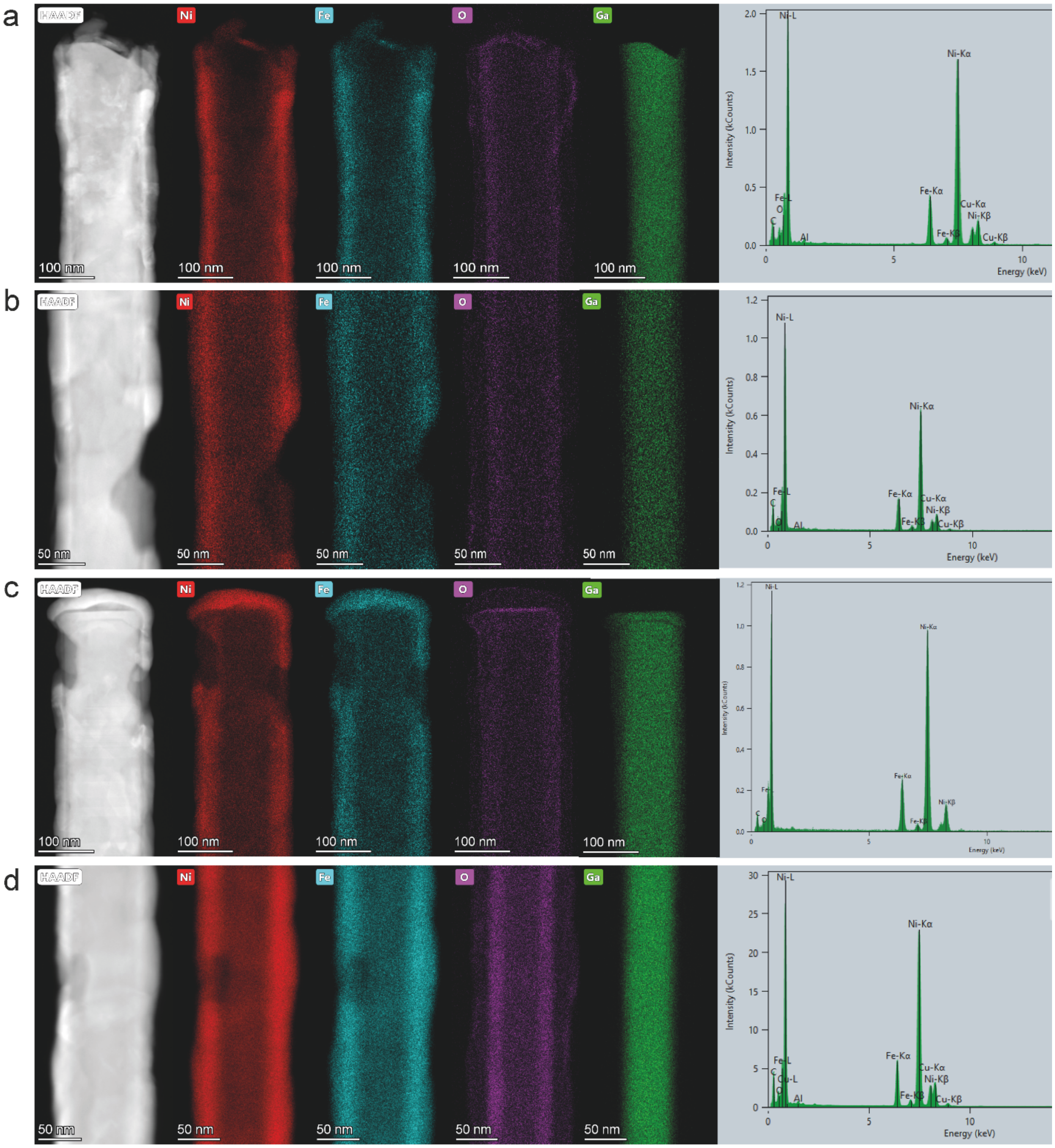

Figure S3. HADDAF images and elemental maps of NiFe nanotube samples (a-d) NT_S1-4, prepared with Ni:Fe pulse ratio *m* = 6, annealed at 380 ˚C, and corresponding energy dispersive spectroscopy analysis of the external shell. The composition and NiFe shell thickness values measured for the four nanotubes are summarized in Table S1.

Table S1. Thickness and compositional values of NiFe NTs prepared by ALD with Ni:Fe pulse ratio *m* = 6. The values were extrapolated from STEM-EDX analysis.

Sample	Thickness far from holes (nm)	Ni (at%) in NiFe ratio	Fe (at%) in NiFe ratio	O (at%) in the shell
NT1 (main text)	21.1	80.2	19.8	3.5
NT_S1	23.3	80.7	19.3	3.4
NT_S2	20.2	80.5	19.5	3.6
NT_S3	23.3	80.7	19.3	3.5
NT_S4	22.1	80.8	19.2	3.5
**Average**	21.6 ± 1.2	80.4 ± 0.3	19.6 ± 0.3	3.5 ± 0.1

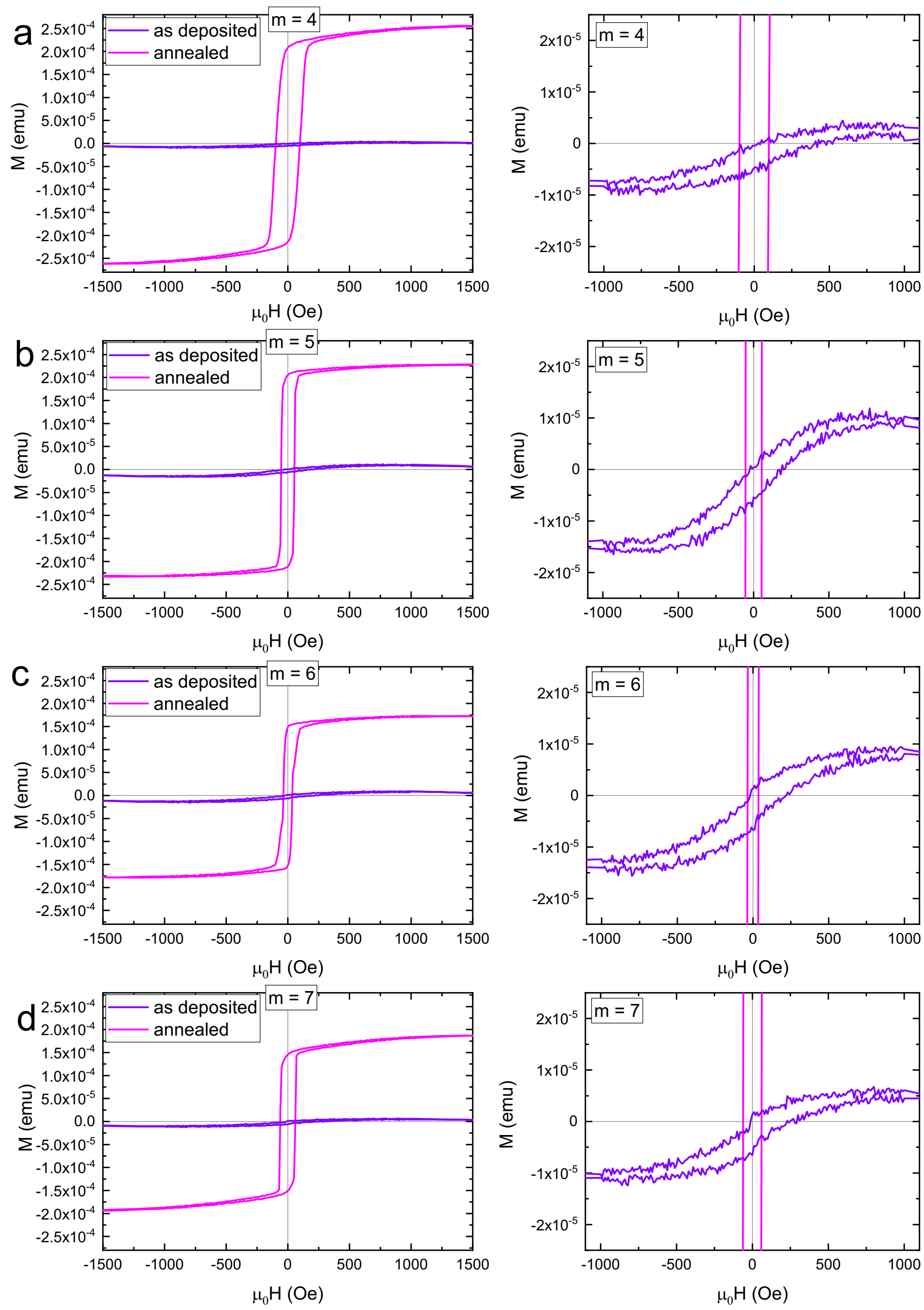

Figure S4. Hysteresis curves measured with a VSM, at room temperature, of as deposited (purple curve) and annealed (magenta curve) NiFe thin films prepared by ALD, using Ni:Fe pulse ratio (a) *m* = 4, (b) *m* = 5 , (c) *m* = 6 and (d) *m* = 7. The hysteresis curves were acquired with an applied field oriented at 0˚ degrees in the plane of the sample. On the right panel the same curves are magnified.

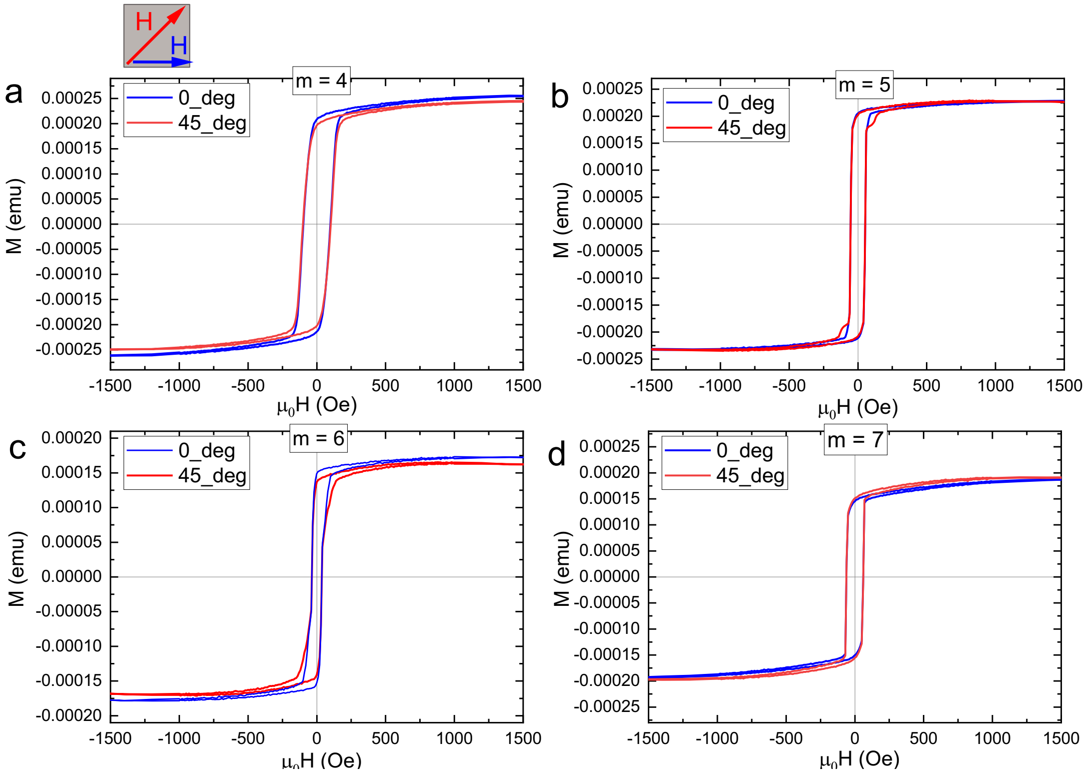

Figure S5. Hysteresis curves measured with a VSM at room temperature of annealed NiFe thin films prepared by ALD, using Ni:Fe pulse ratio (a) *m* = 4, (b) *m* = 5 , (c) *m* = 6 and (d) *m* = 7. The blue (red) curves were acquired with an applied field oriented at 0˚ (45) degrees in the plane of the sample.

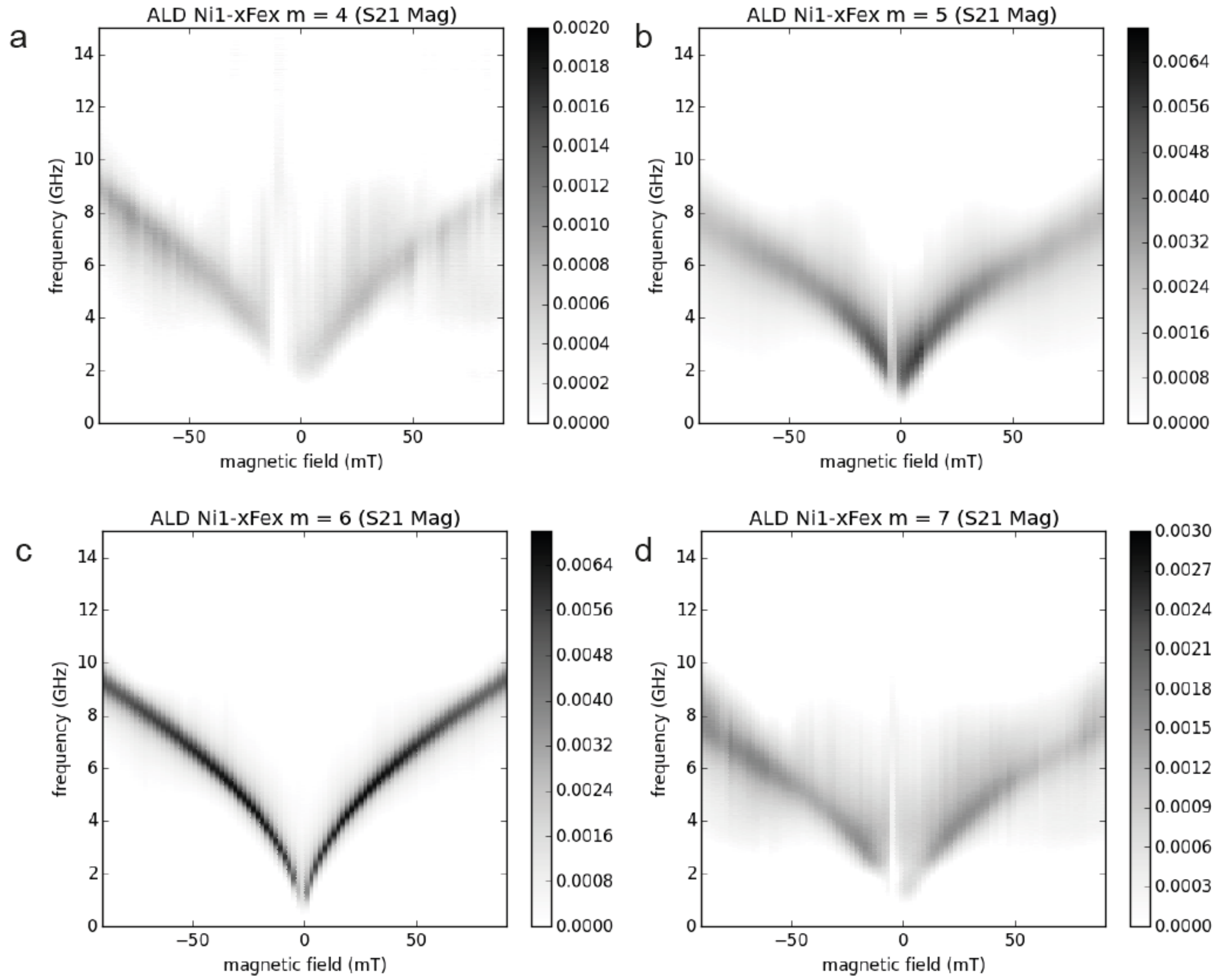

Figure S6. VNA-FMR measurements of annealed NiFe thin films prepared by ALD, using Ni:Fe pulse ratio (a) *m* = 4, (b) *m* = 5 , (c) *m* = 6 and (d) *m* = 7. The resonance measurements were acquired while sweeping the magnetic field from 90 to -90 mT in the plane of the sample.

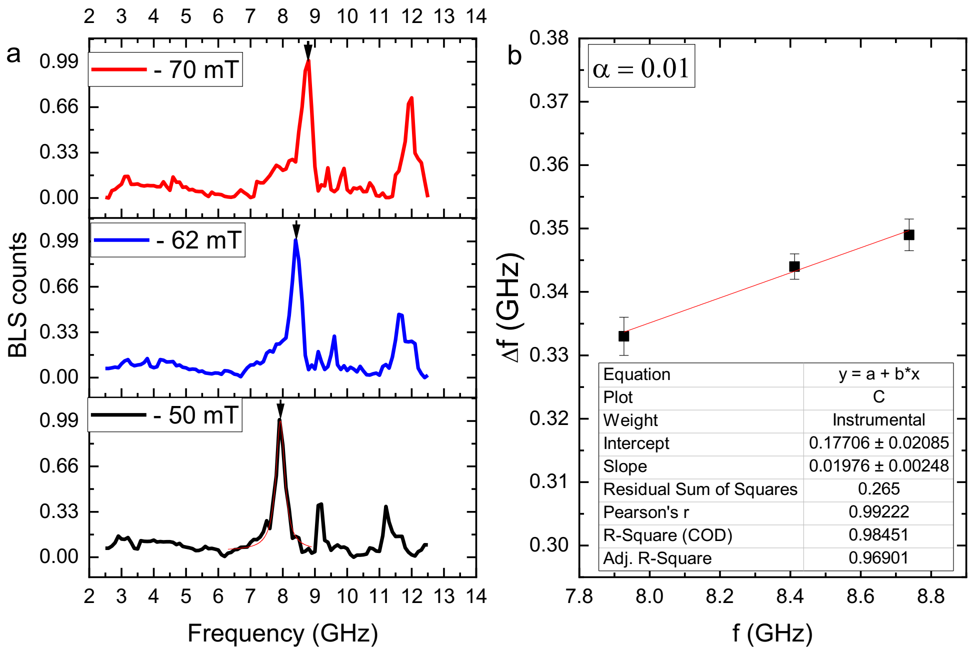

Figure S7. (a) BLS spectra detected, at different applied static magnetic fields, on an individual permalloy NT prepared by PEALD using Ni : Fe pulse ratio *m* = 6. Black arrows indicate the peak (eigenmode) whose linewidth was used to assess the NT damping parameter. (b) Linewidth Δf plotted as function of the resonance frequency of the mode selected. The data are fitted with a linear function whose slope is twice the Gilbert damping parameter α, that is found to be 0.01 consistent with the VNA-FMR data.
